# Immune dysfunction and food-specific IgG associated erosive oral lichen planus: a two-hit pathogenic model

**DOI:** 10.3389/fimmu.2026.1804758

**Published:** 2026-05-11

**Authors:** Yanjie Yang, Lijie Yang, Fei Liu, Guangyan Cheng, Qigen Fang

**Affiliations:** 1 Department of Stomatology, The First Affiliated Hospital of Zhengzhou University, Zhengzhou, China; 2 Department of Head Neck, The Affiliated Cancer Hospital of Zhengzhou University & Henan Cancer Hospital, Zhengzhou, China

**Keywords:** cellular immunity, erosive phenotype, food-specific IgG, oral lichen planus, systemic inflammation

## Abstract

**Background:**

Oral lichen planus (OLP) is a chronic immune-mediated mucosal disorder with a heterogeneous clinical course and potential for malignant transformation. While T-cell dysfunction is central to its pathogenesis, the role of humoral immune responses, particularly food-specific IgG-mediated reactions, remains underexplored. This study aimed to comprehensively evaluate the cellular and humoral immune profiles in OLP patients and investigate their association with clinical phenotypes and inflammatory biomarkers.

**Methods:**

In this retrospective cross-sectional study, 696 OLP patients were enrolled. Peripheral lymphocyte subsets (CD3^+^, CD4^+^, CD8^+^, CD19^+^, NK cells) were quantified by flow cytometry. Serum levels of food-specific IgG antibodies against 14 common antigens and inflammatory mediators (CRP, IL-6, TNF-α, IFN-γ, IL-17A) were measured by ELISA. Patients were stratified by age, gender, and clinical type (erosive vs. non-erosive). Statistical analyses included correlation tests, logistic regression, and subgroup profiling based on immune status and IgG positivity.

**Results:**

OLP patients exhibited significant suppression of total T, helper T, cytotoxic T, B, and NK cells compared to healthy references. Food-specific IgG antibodies were detected in 57.6% of patients, with eggs being the most prevalent trigger. Female patients showed higher IgG positivity and elevated IL-6/IL-17A levels. Erosive OLP was independently associated with longer disease duration, lower CD3^+^/CD4^+^ T-cell counts, and higher IL-6, TNF-α, and IL-17A. IgG positivity was predicted by female gender, low CD3^+^/CD4^+^ counts, high B/NK cells, and elevated IL-6, IL-17A, and CRP. Subgroup analysis identified a high-risk phenotype (immune-suppressed + IgG-positive) with the most severe erosive disease, highest inflammation, and multiple-food reactivity.

**Conclusion:**

Our findings suggest a distinct immune-dietary interplay in OLP, where T-cell deficiency and food-specific IgG responses are closely linked to systemic inflammation and erosive disease. This supports a “Two-Hit” pathogenic model and highlights the potential for combined immunomodulatory and dietary interventions in high-risk patients.

## Introduction

Oral lichen planus (OLP) is a chronic immune-mediated mucosal disorder characterized by a heterogeneous clinical course and a well-documented potential for malignant transformation. The clinical presentation of OLP varies significantly, ranging from asymptomatic reticular patterns to severe, painful erosive forms (1). While the precise etiology remains elusive, the prevailing understanding of OLP pathogenesis centers on T-cell dysfunction. Historically, the disease has been defined by a dense sub-epithelial infiltrate of T-lymphocytes, leading to basal keratinocyte destruction (2). However, reliance on a purely cellular immune model fails to fully account for the systemic inflammatory nature of the condition or the wide variability in clinical phenotypes, particularly the distinction between erosive and non-erosive forms (3). Consequently, the role of humoral immune responses, particularly food-specific IgG-mediated reactions, remains a critical yet underexplored frontier in OLP research.

Emerging evidence suggests that the mucosal immune system is not isolated but functions within a broader systemic context involving dietary interactions and inflammatory mediators (4). Unlike immediate IgE-mediated allergies, food-specific IgG antibodies trigger immune complex-mediated reactions, often resulting in the formation and deposition of circulating immune complexes (5). In conditions of immune dysregulation, these complexes may act as chronic inflammatory triggers (6). Despite this, the association between specific dietary antigens and oral mucosal disease activity has not been rigorously characterized. This study posits a “Two-Hit” pathogenic model, hypothesizing that an initial systemic cellular immune dysregulation—specifically T-cell deficiency—compromises the mucosal barrier, thereby facilitating the translocation of dietary antigens. This breach ostensibly provokes a secondary adaptive response characterized by high titers of food-specific IgG, potentially contributing to a systemic inflammatory response dominated by cytokines such as interleukin-6 (IL-6) and interleukin-17A (IL-17A).

To validate this hypothesis and identify high-risk clinical profiles, this study aimed to comprehensively evaluate the cellular and humoral immune profiles in a large cohort of OLP patients and investigate their association with clinical phenotypes and inflammatory biomarkers. In this retrospective cross-sectional analysis of 696 patients, we quantified peripheral lymphocyte subsets—including CD3^+^, CD4^+^, CD8^+^, CD19^+^, and Natural Killer (NK) cells—to assess cellular immunity. Concurrently, we measured serum levels of food-specific IgG antibodies against 14 common antigens, including eggs, wheat, and soybeans, to map the prevalence and severity of dietary intolerance.

Furthermore, to establish the link between immune-dietary interactions and disease severity, we analyzed key inflammatory mediators, including C-reactive protein (CRP), TNF-α, IFN-γ, IL-6, and IL-17A. By stratifying patients based on age, gender, and clinical type, this research seeks to isolate independent predictors of the erosive phenotype. Our findings aim to reveal a distinct immune-dietary interplay in OLP, highlighting how T-cell deficiency and food-specific IgG responses are synergistically associated with systemic inflammation, thereby supporting the rationale for combined immunomodulatory and dietary interventions in the management of high-risk patients.

## Methods

### Study design and population

This retrospective cross-sectional study included patients diagnosed with OLP who visited the Department of Oral Mucosal Diseases, The First Affiliated Hospital of Zhengzhou University, between January 2017 and December 2025. Patients were categorized into three age groups according to the World Health Organization (WHO) classification: young adults (18–44 years), middle-aged adults (45–59 years), and elderly adults (≥60 years). The study was conducted in accordance with the principles of the Declaration of Helsinki and was approved by the Institutional Review Board of The First Affiliated Hospital of Zhengzhou University.

### Inclusion and exclusion criteria

The inclusion criteria comprised the following: (1) a clinical and/or histopathological diagnosis of OLP; (2) a disease duration exceeding three months; (3) an age of 18 years or older; and (4) the availability of complete clinical and laboratory records. Participants were excluded based on the following criteria: (1) the coexistence of other autoimmune diseases (such as systemic lupus erythematosus or rheumatoid arthritis), infectious diseases, or malignancies; (2) the presence of severe systemic disorders (including renal failure or hepatic dysfunction); (3) pregnancy or lactation; (4) malnutrition or documented eating disorders; (5) the use of systemic or topical immunosuppressive agents (for example, corticosteroids or calcineurin inhibitors) or immunomodulators within the 30 days preceding the study; and (6) recent antibiotic or probiotic use within the two weeks prior to blood sampling.

A healthy control group was recruited from individuals who underwent routine health check-ups at the same hospital during the study period. Controls were selected to match the OLP cohort in terms of age (± 3 years) and sex distribution. Inclusion criteria for controls were: (1) absence of any oral mucosal disease confirmed by clinical examination; (2) no history of autoimmune, inflammatory, or malignant diseases; (3) no use of immunosuppressive or immunomodulatory agents within the past three months; and (4) no acute or chronic infections. Controls with a history of allergic diseases, gastrointestinal disorders, or recent antibiotic/probiotic use were excluded.

### Clinical data and sample collection

Demographic and clinical data, including age, gender, disease duration, and clinical phenotype (erosive vs. non-erosive), were extracted from electronic medical records. Peripheral venous blood (5 mL) was collected from each participant after an overnight fast (≥8 hours). Samples were drawn into EDTA-anticoagulant tubes for flow cytometry and into serum separation tubes for IgG antibody testing. All samples were processed within 4 hours of collection.

### Cellular immune function

Cellular immune function was assessed through the quantification of lymphocyte subsets via four-color flow cytometry (Beckman Coulter, USA). The specific subsets analyzed included: total T lymphocytes (T#: CD3^+^), helper/inducer T cells (CD3^+^CD4^+^), suppressor/cytotoxic T cells (CD3^+^CD8^+^), B lymphocytes (B#: CD19^+^), and Natural Killer cells (NK#: CD3^-^CD16^+^CD56^+^). Staining was performed using commercial antibody cocktails (specifically, CD3/CD8/CD45/CD4 and CD3/CD16+CD56/CD45/CD19 from Beckman Coulter). Absolute cell counts were subsequently calculated according to the laboratory’s standardized protocol. These counts were then compared against internal reference ranges, which were established from a validated cohort of 200 healthy donors within the clinical laboratory.

### Detection of food-specific IgG antibodies

Serum levels of IgG antibodies against 14 common food antigens—namely beef, chicken, cod, corn, crab, eggs, mushrooms, milk, pork, rice, shrimp, soybeans, tomatoes, and wheat—were measured using a commercial enzyme-linked immunosorbent assay (ELISA) kit (Jiangsu Haooubo Biopharmaceutical Co., Ltd., China) in strict accordance with the manufacturer’s instructions. The positivity threshold for these antibodies was defined as a concentration exceeding 49 U/mL. Positive results were further graded in severity as mild (+1) for levels between 50 and 99 U/mL, moderate (+2) for 100 to 199 U/mL, and severe (+3) for levels of 200 U/mL or greater. Finally, multiple-food positivity was defined as the presence of IgG antibodies against two or more of the tested food antigens.

### Inflammatory biomarker assay

Serum levels of key inflammatory mediators were measured using commercially available enzyme-linked immunosorbent assay (ELISA) kits (R&D Systems, USA), strictly following the manufacturer’s protocols. The selected biomarkers included C-reactive protein (CRP) as a global marker of systemic inflammatory burden; the pro-inflammatory cytokines interleukin-6 (IL-6) and tumor necrosis factor-alpha (TNF-α), which are central to immune activation; as well as T-helper cell polarization markers—interferon-gamma (IFN-γ) as a representative Th1-type cytokine and interleukin-17A (IL-17A) as a representative Th17-type cytokine.

### Statistical analysis

Data were analyzed using R (version 4.5.2), two-tailed p-values < 0.05 were considered statistically significant. Continuous variables (immune cell counts, cytokine levels) were expressed as mean ± standard deviation (SD) and compared using independent samples t-tests or one-way ANOVA followed by Tukey’s *post-hoc* test. Categorical variables (IgG positivity rates, clinical phenotypes) were presented as frequencies and percentages, and compared using Chi-square or Fisher’s exact test as appropriate. Pearson or Spearman correlation analysis was used to assess the relationships between lymphocyte subset counts and food-specific IgG antibody levels, as well as between inflammatory markers and immune/clinical parameters.

To identify independent factors associated with food intolerance and clinical phenotypes, binary logistic regression and multivariate linear regression models were employed, with age, gender, clinical type, and immune parameters included as covariates. Patients were further stratified into four subgroups based on immune status (suppressed vs. normal) and food IgG positivity, and group comparisons were performed to identify distinct immune-dietary profiles.

## Results

### Baseline data

A total of 696 eligible OLP patients were included in this study. The mean age of the cohort was 48.7 ± 12.3 years (range: 18–78 years). According to the WHO age classification, 298 cases (42.8%) were categorized as young adults (18–44 years), 267 cases (38.4%) as middle-aged adults (45–59 years), and 131 cases (18.8%) as elderly adults (≥60 years). There were 245 male patients (35.2%) and 451 female patients (64.8%), yielding a male-to-female ratio of 1:1.84. The mean disease duration was 28.4 ± 18.6 months. Clinically, 347 patients (49.9%) presented with the erosive form of OLP (E-OLP), while 349 patients (50.1%) had the non-erosive form (NE-OLP), which included reticular, plaque-like, and other subtypes. Regarding lesion distribution, the buccal mucosa was the most commonly affected site (85.2%), followed by the tongue (45.1%), gingiva (32.8%), and other sites (15.5%); note that some patients exhibited lesions at multiple sites (**Table 1**).

**Table 1:** Baseline demographic and clinical characteristics of Oral lichen planus (OLP) patients (n=696).

Characteristic	Total (n=696)	Erosive OLP (n=347)	Non-Erosive OLP (n=349)	p
Age (years), Mean ± SD	48.7 ± 12.3	49.1 ± 11.9	48.3 ± 12.7	0.352
Age Group, n (%)				0.421
Young (18-44 yrs)	298 (42.8)	142 (40.9)	156 (44.7)	
Middle-aged (45-59 yrs)	267 (38.4)	139 (40.1)	128 (36.7)	
Elderly (≥60 yrs)	131 (18.8)	66 (19.0)	65 (18.6)	
Gender, n (%)				0.215
Male	245 (35.2)	115 (33.1)	130 (37.2)	
Female	451 (64.8)	232 (66.9)	219 (62.8)	
Disease Duration (months), Mean ± SD	28.4 ± 18.6	30.1 ± 19.2	26.7 ± 17.9	0.014*
Lesion Site, n (%)†				
Buccal Mucosa	593 (85.2)	301 (86.7)	292 (83.7)	0.265
Tongue	314 (45.1)	170 (49.0)	144 (41.3)	0.041*
Gingiva	228 (32.8)	105 (30.3)	123 (35.2)	0.172

SD, Standard Deviation.

†, Percentages may sum to >100% as some patients had multiple lesion sites.

*, Statistically significant difference (P < 0.05).

The healthy control group (n=200) was well-matched to the OLP patient cohort in terms of age and sex (**Supplementary Table 1**).

### Cellular immune function assessment

Compared to established laboratory reference ranges, the mean absolute counts of total T lymphocytes (1050 ± 320 cells/μL vs. ref: 1200–1800, p < 0.001), helper T cells (620 ± 210 cells/μL vs. ref: 700–1100, p < 0.001), cytotoxic T cells (410 ± 185 cells/μL vs. ref: 500–900, p < 0.001), B lymphocytes (180 ± 85 cells/μL vs. ref: 200–400, p < 0.001), and NK cells (150 ± 70 cells/μL vs. ref: 200–500, p < 0.001) were consistently and significantly lower in OLP patients. In further comparison with age-matched and sex-matched healthy controls. Across all five strata, OLP patients exhibited significantly lower absolute counts of CD3^+^ T cells, CD4^+^ T cells, CD8^+^ T cells, CD19^+^ B cells, and NK cells (**Supplementary Tables 2**, **3**, all p < 0.01), confirming that the observed cellular immune deficiencies represent a disease-associated abnormality rather than an artifact of age distribution.

Significant age-related variations were observed for specific subsets. The absolute count of CD8^+^ T cells was lowest in the middle-aged group (45–59 years: 380 ± 175 cells/μL) compared to both the young adult (18–44 years: 440 ± 190 cells/μL, p=0.003) and elderly groups (≥60 years: 430 ± 195 cells/μL, p=0.028). Conversely, NK cell counts exhibited a progressive increase with age, with the elderly group demonstrating significantly higher levels (185 ± 75 cells/μL) than both the young (135 ± 65 cells/μL, p<0.001) and middle-aged groups (145 ± 68 cells/μL, p<0.001).

Gender-specific disparities were evident within age strata. Among young adults (18–44 years), female patients had significantly lower counts of total T cells (980 ± 305 vs. 1180 ± 335 cells/μL in males, p=0.001), B cells (165 ± 80 vs. 210 ± 90 cells/μL, p=0.004), and NK cells (120 ± 60 vs. 160 ± 72 cells/μL, p=0.002). No statistically significant gender differences were found in the middle-aged group. In the elderly cohort (≥60 years), however, female patients displayed higher counts of CD4^+^ T cells (680 ± 220 vs. 580 ± 200 cells/μL in males, p=0.012) and B cells (210 ± 90 vs. 155 ± 80 cells/μL, p=0.007).

**Figure 1:**
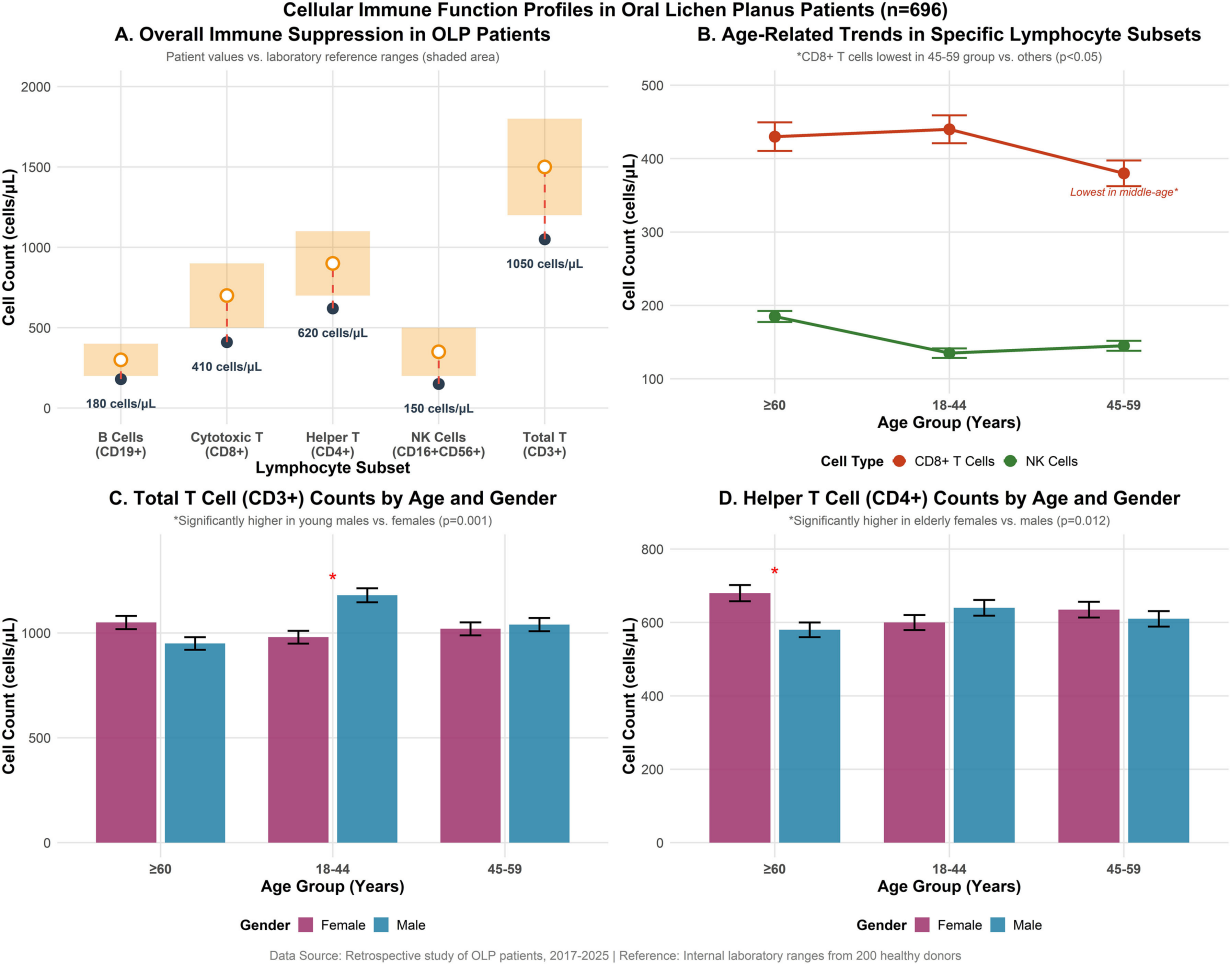
Cellular immune function assessment in 696 oral lichen planus (OLP) patients. **(A)** Overall suppression of lymphocyte subsets compared to reference ranges. **(B)** Age-related trends showing CD8^+^ T cells lowest in middle age and NK cells increasing with age. **(C)** Gender differences in total T cells, with young males having higher counts. **(D)** Gender differences in CD4^+^ T cells, with elderly females having higher counts. Error bars represent standard deviation. *p<0.05.

Longitudinal analysis revealed distinct immune aging trajectories between genders. In male patients, a general decline in immune cell counts was observed with advancing age. Total T cell counts were significantly higher in the youth group compared to the elderly group (1180 ± 335 vs. 950 ± 300 cells/μL, p<0.001), and B cell counts also decreased significantly from youth to elderly (210 ± 90 vs. 155 ± 80 cells/μL, p=0.005). In contrast, female patients showed an increasing trend in specific immune markers. Counts of CD4^+^ T cells (600 ± 205 to 680 ± 220 cells/μL, p=0.009) and total T cells (980 ± 305 to 1050 ± 315 cells/μL, p=0.038) were significantly higher in the elderly compared to the young group. Furthermore, NK cell counts in females increased progressively across all three age groups (120 ± 60, 150 ± 70, and 190 ± 78 cells/μL, respectively, with all inter-group comparisons p<0.05) (**Figure 1**).

### Serum food-specific IgG antibody detection

**Figure 2:**
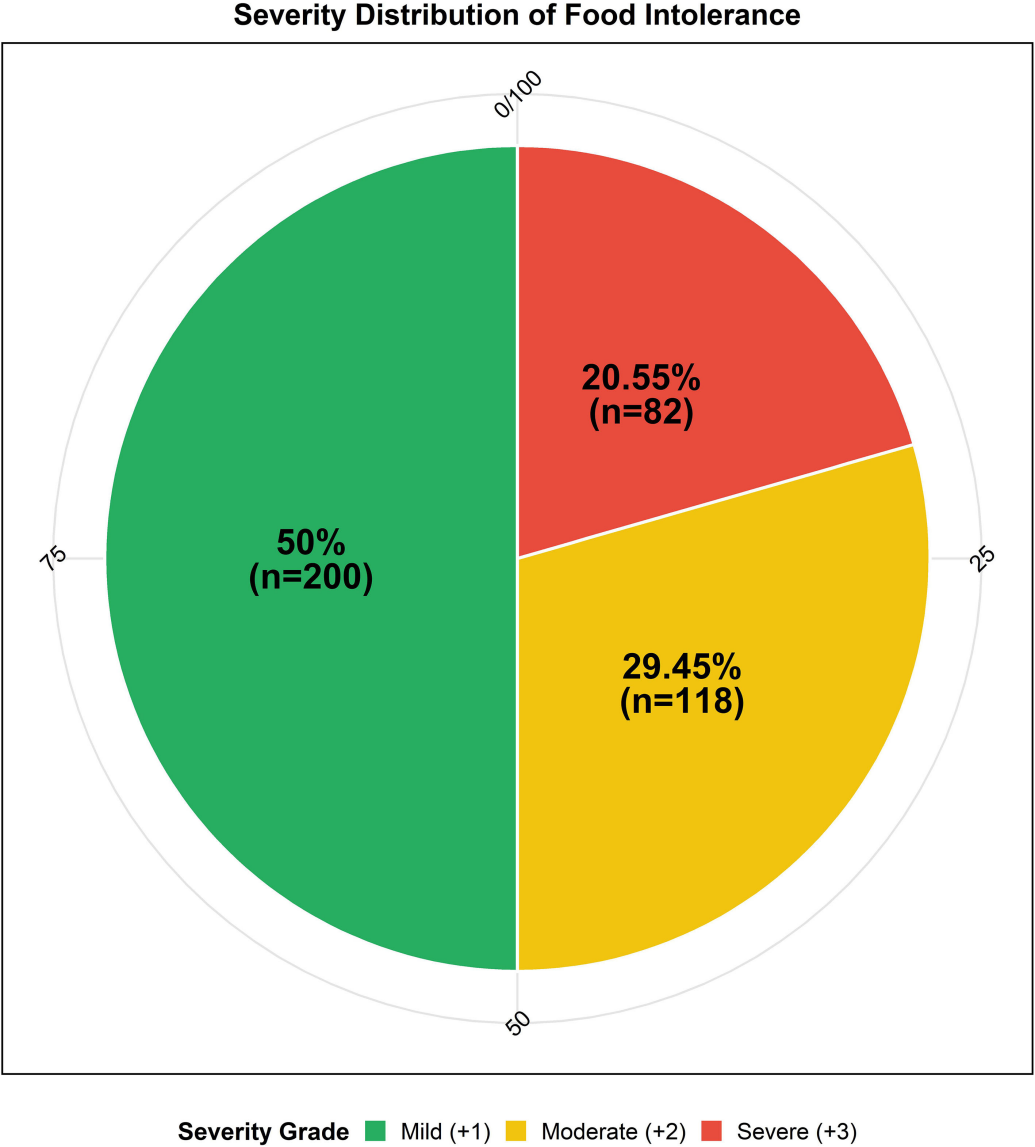
Severity distribution of food intolerance among IgG-positive oral lichen planus (OLP) patients.

**Figure 3:**
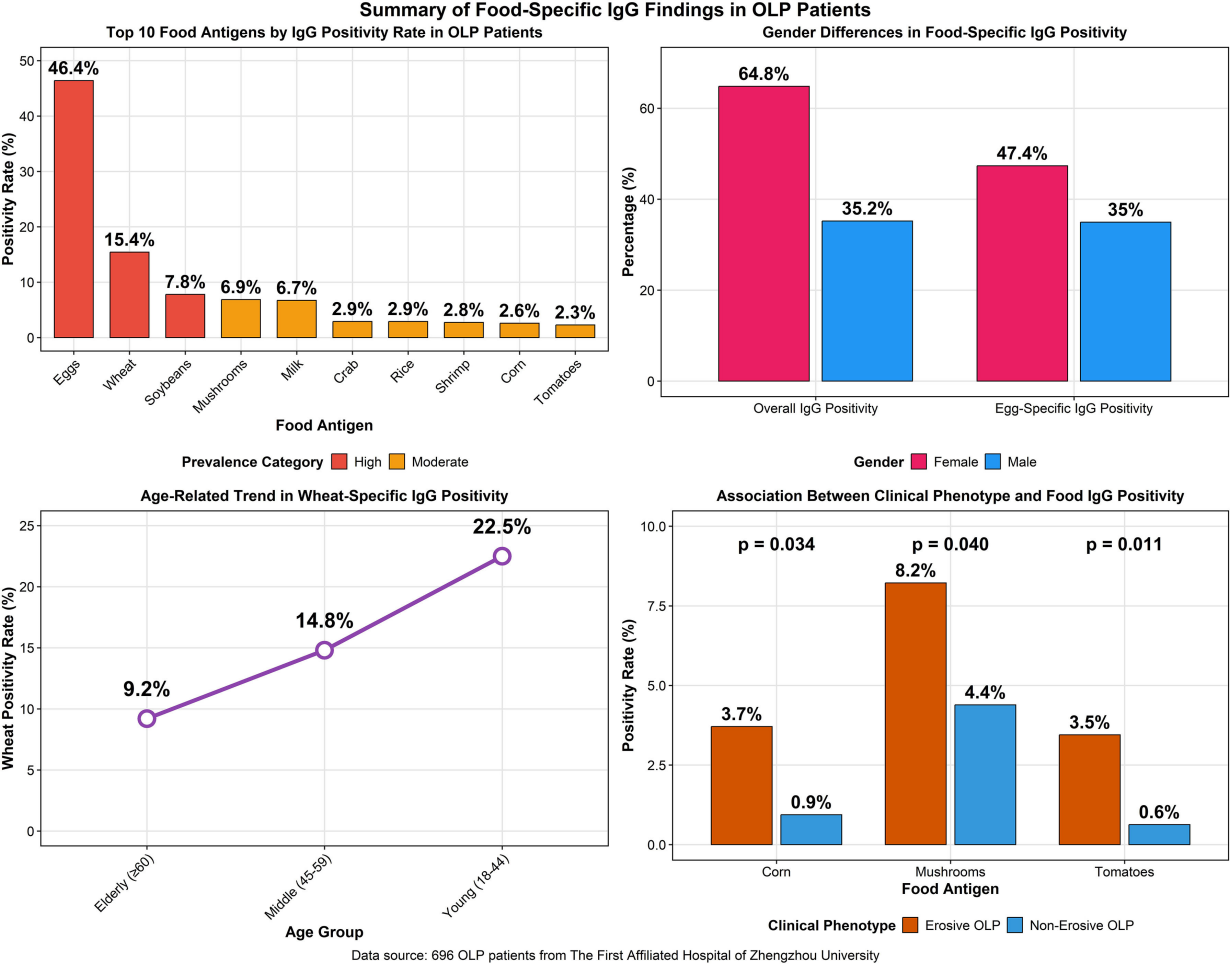
Multi-panel summary of key food-specific IgG findings in oral lichen planus (OLP) patients, including antigen prevalence, demographic variations, and clinical associations.

The detection of serum food-specific IgG antibodies revealed that 57.6% (401/696) of OLP patients were positive. Among the positive cases, 50.0% exhibited mild positivity (+1), 29.5% were moderate (+2), and 20.5% were severe (+3). Multiple-food positivity, defined as reactivity to two or more antigens, was observed in 35.66% of positive individuals. When ranked by prevalence, eggs were the most common trigger (46.41%), followed by wheat (15.42%) and soybeans (7.79%); other foods such as mushrooms, milk, rice, and seafood showed moderate to low positivity rates. Significant gender-based differences were noted, with female patients demonstrating a higher overall positivity rate (p = 0.008) and a specifically elevated reactivity to eggs (47.35% vs. 34.95% in males, p = 0.003). Age-related analysis indicated that wheat intolerance was significantly more prevalent in younger adults and decreased with advancing age (p = 0.008), while no significant age-related variations were observed for eggs or soybeans. Furthermore, when comparing clinical phenotypes, patients with erosive OLP showed significantly higher positivity rates to tomatoes, corn, and mushrooms compared to those with non-erosive disease (p < 0.05 for each). In contrast, no significant associations were found between clinical phenotype and intolerance to eggs, wheat, milk, or seafood antigens (**Figures 2**, **3**).

Compared with age- and sex-matched healthy controls, OLP patients demonstrated a significantly higher overall prevalence of food-specific IgG positivity (57.6% vs. 34.5%, p < 0.001). Multiple-food positivity (reactivity to ≥2 antigens) was also more frequent in OLP patients, affecting 20.5% of the total OLP cohort compared with 6.5% of controls (p < 0.001). Among healthy controls, the most commonly detected food antigens were eggs (24.0%), wheat (12.5%), and milk (9.5%), which mirrored the rank order observed in OLP patients but with consistently lower prevalence. Gender-based differences in IgG positivity were not statistically significant in the healthy control group, contrasting with the significant female predominance observed in the OLP (**Table 2**).

**Table 2:** Serum inflammatory biomarker levels in OLP patients and healthy controls.

Biomarker	OLP patients (n=696) mean ± SD	Healthy controls (n=200) mean ± SD	p-value
CRP (mg/L)	4.8 ± 2.1	1.2 ± 0.8	<0.001
IL-6 (pg/mL)	12.5 ± 6.3	5.1 ± 2.4	<0.001
TNF-α (pg/mL)	18.2 ± 7.9	8.7 ± 3.5	<0.001
IFN-γ (pg/mL)	25.6 ± 11.4	15.3 ± 6.8	<0.001
IL-17A (pg/mL)	9.8 ± 4.5	3.2 ± 1.6	<0.001
Any food-specific lgG positivity, n (%)	401 (57.6)	69 (34.5)	<0.001
Multiple-food positivity, n (%)	143 (20.5)	13 (6.5)	<0.001

### Inflammatory biomarker assay

Serum levels of all measured inflammatory mediators were significantly elevated in OLP patients compared to healthy controls (p < 0.001). CRP, IL-6, TNF-α, IFN-γ, and IL-17A were markedly higher in the patient cohort. The most pronounced elevations were observed for the cytokines IL-6 and IL-17A, which were approximately 2.5-fold and 3.1-fold higher than in controls, respectively (**Table 2**).

Elderly patients (≥60 years) exhibited significantly higher levels of CRP (5.9 ± 2.3 mg/L) and IL-6 (14.8 ± 6.8 pg/mL) compared to both young (18–44 yrs) and middle-aged (45–59 yrs) groups (p < 0.05 for all comparisons). In contrast, levels of IFN-γ and IL-17A did not show significant variation across age groups.

Female patients demonstrated moderately but significantly higher serum levels of IL-6 (13.4 ± 6.5 pg/mL vs. 11.1 ± 5.8 pg/mL in males, p=0.007) and IL-17A (10.5 ± 4.7 pg/mL vs. 8.7 ± 4.1 pg/mL, p=0.002). No significant gender differences were observed for CRP, TNF-α, or IFN-γ.

Patients with E-OLP displayed a more pronounced pro-inflammatory state. Serum levels of IL-6 (14.2 ± 6.9 pg/mL vs. 10.8 ± 5.4 pg/mL, p<0.001), TNF-α (20.1 ± 8.3 pg/mL vs. 16.3 ± 7.1 pg/mL, p<0.001), and IL-17A (11.2 ± 4.8 pg/mL vs. 8.4 ± 3.9 pg/mL, p<0.001) were all significantly higher in the E-OLP group compared to the NE-OLP group. CRP and IFN-γ levels were also elevated in E-OLP, but the differences did not reach statistical significance.

### Correlation analysis

Serum IL-6 levels showed a significant negative correlation with absolute counts of CD4^+^ T cells (r = -0.18, p=0.001) and total T cells (r = -0.15, p=0.005). IL-17A levels were positively correlated with NK cell counts (r = 0.21, p<0.001). No significant correlations were found between inflammatory markers and B cell or CD8^+^ T cell counts.

Patients who were positive for food-specific IgG antibodies (n=401) had significantly higher median levels of CRP (5.2 mg/L vs. 4.3 mg/L, p=0.013) and IL-6 (13.8 pg/mL vs. 11.0 pg/mL, p=0.003) compared to IgG-negative patients. Furthermore, a positive correlation was observed between the number of reactive food antigens (multiple-food positivity) and serum IL-6 levels (r = 0.24, p<0.001). Subgroup analysis indicated that patients with severe food intolerance (+3 grade) exhibited the highest levels of IL-17A (12.1 ± 5.2 pg/mL, p=0.009 for trend).

Lower absolute counts of total T cells (CD3^+^, ρ = -0.18, p = 0.001) and helper T cells (CD4^+^, ρ = -0.21, p < 0.001) were weakly but significantly associated with higher total food-specific IgG levels. Similarly, a negative correlation was observed between these T-cell subsets and the number of reactive food antigens (total T cells: ρ = -0.15, p = 0.004; CD4^+^ T cells: ρ = -0.19, p = 0.001). In contrast, B lymphocyte (CD19^+^) counts showed a weak positive correlation with both total IgG levels (ρ = 0.12, p = 0.018) and the number of reactive foods (ρ = 0.10, p = 0.039). Natural killer (NK) cell counts were also positively correlated with IgG-related parameters (total IgG: ρ = 0.16, p = 0.003; number of reactive foods: ρ = 0.13, p = 0.011). No significant correlation was found between cytotoxic T cell (CD8^+^) counts and food-specific IgG measures.

### Risk factors for erosive OLP

Univariate analysis revealed that longer disease duration (OR = 1.01, p=0.015), reduced total T-cell (CD3^+^; OR = 0.68, p<0.001) and helper T-cell (CD4^+^; OR = 0.64, p<0.001) counts, as well as elevated serum levels of IL-6 (OR = 1.11, p<0.001), TNF-α (OR = 1.07, p<0.001), and IL-17A (OR = 1.13, p<0.001) were significantly associated with erosive OLP. Age, gender, cytotoxic T-cell (CD8^+^), B-cell, and NK-cell counts, along with CRP and IFN-γ levels, were not significant in univariate screening. In the multivariable model adjusted for all listed variables, disease duration (OR = 1.01, p=0.014), low CD3^+^ (OR = 0.72, p<0.001) and CD4^+^ T-cell counts (OR = 0.68, p<0.001), and high IL-6 (OR = 1.10, p<0.001), TNF-α (OR = 1.07, p<0.001), and IL-17A (OR = 1.12, p<0.001) remained independent predictors of erosive disease (**Table 3**).

**Table 3:** Risk factors for erosive oral lichen planus (OLP).

Variable	Univariate		Multivariable	
	OR (95% CI)	p-value	OR (95% CI)	p-value
Age (years)	1.01 (0.99–1.02)	0.121	1.01 (0.99–1.03)	0.352
Gender (Female vs. Male)	1.20 (0.90–1.61)	0.215	1.22 (0.89–1.67)	0.215
Disease Duration (months)	1.01 (1.00–1.02)	0.015*	1.01 (1.00–1.02)	0.014*
Total T cells (CD3^+^)	0.68 (0.57–0.82)	<0.001*	0.72 (0.60–0.86)	<0.001*
Helper T cells (CD4^+^)	0.64 (0.53–0.77)	<0.001*	0.68 (0.55–0.84)	<0.001*
Cytotoxic T cells (CD8^+^)	0.94 (0.82–1.09)	0.432	0.95 (0.82–1.10)	0.491
B cells (CD19^+^)	1.18 (0.99–1.41)	0.065	1.15 (0.96–1.38)	0.124
NK cells	1.06 (0.91–1.23)	0.458	1.08 (0.92–1.27)	0.346
IL-6 (pg/mL)	1.11 (1.06–1.16)	<0.001*	1.10 (1.05–1.15)	<0.001*
TNF-α (pg/mL)	1.07 (1.03–1.11)	<0.001*	1.07 (1.03–1.11)	<0.001*
IL-17A (pg/mL)	1.13 (1.08–1.18)	<0.001*	1.12 (1.06–1.18)	<0.001*
CRP (mg/L)	1.06 (0.98–1.15)	0.148	1.05 (0.97–1.14)	0.198
IFN-γ (pg/mL)	1.02 (1.00–1.05)	0.078	1.02 (0.99–1.05)	0.172

OR, Odds Ratio; CI, Confidence Interval.

Multivariate model adjusted for age, gender, disease duration, and all listed immune/inflammatory parameters.

*Statistically significant (p < 0.05).

### Predictors of food-specific IgG positivity

Univariate analysis indicated that female gender (OR = 1.48, p=0.004), lower total T-cell (CD3^+^; OR = 0.76, p=0.001) and helper T-cell (CD4^+^; OR = 0.70, p<0.001) counts, higher B-cell (OR = 1.22, p=0.024) and NK-cell (OR = 1.26, p=0.006) counts, and elevated serum levels of IL-6 (OR = 1.09, p<0.001), IL-17A (OR = 1.07, p=0.005), and CRP (OR = 1.13, p=0.010) were significantly associated with food IgG positivity. Age, clinical phenotype (erosive vs. non-erosive), cytotoxic T-cell (CD8^+^) count, TNF-α, and IFN-γ levels were not significant. In the multivariable model adjusting for all covariates, female gender (OR = 1.45, p=0.008), low CD3^+^ (OR = 0.78, p=0.003) and CD4^+^ T-cell counts (OR = 0.71, p<0.001), high B-cell (OR = 1.25, p=0.018) and NK-cell counts (OR = 1.30, p=0.003), and elevated IL-6 (OR = 1.08, p<0.001), IL-17A (OR = 1.06, p=0.021), and CRP (OR = 1.12, p=0.013) remained independent predictors (**Table 4**).

**Table 4:** Predictors of food-specific IgG positivity.

Variable	Univariate		Multivariable	
	OR (95% CI)	p-value	OR (95% CI)	p-value
Age (years)	0.99 (0.98–1.00)	0.089	0.99 (0.98–1.00)	0.086
Gender (Female vs. Male)	1.48 (1.13–1.94)	0.004*	1.45 (1.10–1.91)	0.008*
Clinical Type (Erosive vs. Non-erosive)	1.22 (0.94–1.58)	0.138	1.18 (0.90–1.55)	0.225
Total T cells (CD3^+^)	0.76 (0.65–0.89)	0.001*	0.78 (0.66–0.92)	0.003*
Helper T cells (CD4^+^)	0.70 (0.59–0.83)	<0.001*	0.71 (0.59–0.86)	<0.001*
Cytotoxic T cells (CD8^+^)	0.99 (0.86–1.15)	0.923	1.01 (0.87–1.17)	0.914
B cells (CD19^+^)	1.22 (1.03–1.45)	0.024*	1.25 (1.04–1.50)	0.018*
NK cells	1.26 (1.07–1.49)	0.006*	1.30 (1.09–1.55)	0.003*
IL-6 (pg/mL)	1.09 (1.05–1.14)	<0.001*	1.08 (1.04–1.13)	<0.001*
TNF-α (pg/mL)	1.03 (0.99–1.07)	0.118	1.02 (0.98–1.06)	0.346
IL-17A (pg/mL)	1.07 (1.02–1.12)	0.005*	1.06 (1.01–1.12)	0.021*
CRP (mg/L)	1.13 (1.03–1.24)	0.010*	1.12 (1.02–1.23)	0.013*
IFN-γ (pg/mL)	1.01 (0.98–1.04)	0.542	1.01 (0.98–1.04)	0.523

OR, Odds Ratio; CI, Confidence Interval.

Multivariate model adjusted for age, gender, clinical type, and all listed immune/inflammatory parameters.

*Statistically significant (p < 0.05).

### Subgroup analysis

Immune suppression defined as total T cells (CD3^+^) <1200 cells/μL; IgG positivity defined as any food-specific IgG ≥50 U/mL. The combined profile of immune suppression and food-specific IgG positivity (Subgroup A) identifies a high-risk phenotype characterized by the oldest age, highest female predominance, longest disease duration, greatest proportion of erosive lesions and tongue involvement, most profound T-cell depletion, elevated B and NK cell counts, the highest levels of pro-inflammatory cytokines (particularly IL-6 and IL-17A), and the most severe and polysensitized food intolerance profile. In contrast, patients with preserved cellular immunity and no detectable food IgG (Subgroup D) represent the mildest clinical spectrum, exhibiting the youngest age, lowest female proportion, shortest disease duration, highest rate of non-erosive disease, normal T-cell counts, lower B/NK cells, and the least systemic inflammation. The intermediate subgroups—immune-suppressed but IgG-negative (Subgroup B) and immune-normal but IgG-positive (Subgroup C)—display graded severities, with Subgroup C showing a preserved cellular compartment but elevated inflammation and moderate food reactivity, while Subgroup B presents with T-cell suppression but lower humoral and inflammatory activation (**Table 5**).

**Table 5:** Comparison of clinical, immune, inflammatory, and IgG profiles across four immune-dietary subgroups.

Parameter/subgroup	Subgroup A: immune-suppressed + IgG-positive (n=251)	Subgroup B: immune-suppressed + IgG-negative (n=185)	Subgroup C: immune-normal + IgG-positive (n=150)	Subgroup D: immune-normal + IgG-negative (n=110)	p (overall)	Post-hoc comparisons (p<0.05)†
Clinical Characteristics						
Age (years, mean ± SD)	50.2 ± 11.8	49.5 ± 12.1	46.1 ± 12.4	46.8 ± 12.9	0.008	A, B > C, D
Female proportion (%)	72.5	68.1	60.0	54.5	0.001	A > C, D; B > D
Erosive type proportion (%)	58.6	52.4	46.7	40.9	0.012	A > C, D
Disease duration (months, mean ± SD)	32.1 ± 19.5	29.8 ± 18.7	26.3 ± 17.1	24.9 ± 16.8	0.005	A > C, D; B > D
Tongue involvement (%)	51.8	48.1	40.7	38.2	0.038	A > C, D
Cellular immune parameters (cells/μL, mean ± SD)						
Total T cells (CD3^+^)	890 ± 245	910 ± 260	1350 ± 290	1380 ± 310	<0.001	A, B < C, D
Helper T cells (CD4^+^)	520 ± 180	540 ± 190	750 ± 210	770 ± 225	<0.001	A, B < C, D
Cytotoxic T cells (CD8^+^)	370 ± 160	380 ± 170	480 ± 195	490 ± 200	<0.001	A, B < C, D
B cells (CD19^+^)	195 ± 80	175 ± 75	205 ± 85	180 ± 78	0.018	A > B; C > D
NK cells (CD3^-^CD16^+^CD56^+^)	165 ± 72	145 ± 68	175 ± 78	150 ± 70	0.009	A > B; C > D
Inflammatory biomarkers (mean ± SD)						
CRP (mg/L)	5.4 ± 2.2	4.9 ± 2.0	5.1 ± 2.1	4.4 ± 1.8	0.002	A > B, D
IL-6 (pg/mL)	14.8 ± 6.9	12.1 ± 5.8	13.5 ± 6.3	10.2 ± 5.1	<0.001	A > B, C, D; C > D
TNF-α (pg/mL)	19.8 ± 8.2	17.9 ± 7.5	18.5 ± 7.9	16.1 ± 7.0	0.001	A > B, D
IL-17A (pg/mL)	11.5 ± 4.9	9.2 ± 4.3	10.8 ± 4.7	8.1 ± 3.8	<0.001	A > B, C, D; C > D
IFN-γ (pg/mL)	26.8 ± 11.9	25.1 ± 11.2	26.2 ± 11.5	23.8 ± 10.7	0.185	NS
Food-specific IgG profiles (IgG-positive subgroups only)‡						
Multiple-food positivity (%)	42.6	–	31.3	–	0.029	A > C
Mean number of reactive foods (mean ± SD)	3.2 ± 1.8	–	2.5 ± 1.5	–	<0.001	A > C
Severe intolerance (+3 grade) proportion (%)	26.3	–	18.0	–	0.048	A > C
Egg reactivity (%)	52.6	–	45.3	–	0.152	NS
Wheat reactivity (%)	18.7	–	14.0	–	0.221	NS

SD, standard deviation; CRP, C-reactive protein; IL, interleukin; TNF-α, tumor necrosis factor-alpha; IFN-γ, interferon-gamma; NK, natural killer; NS, not significant.

†Post-hoc comparisons were performed using Tukey’s test for continuous variables and chi-square with Bonferroni correction for proportions.

‡IgG profiles are only compared between the two IgG-positive subgroups (A, C) because subgroups B and D are IgG-negative by definition.

### Immune-dietary interactions

**Figure 4:**
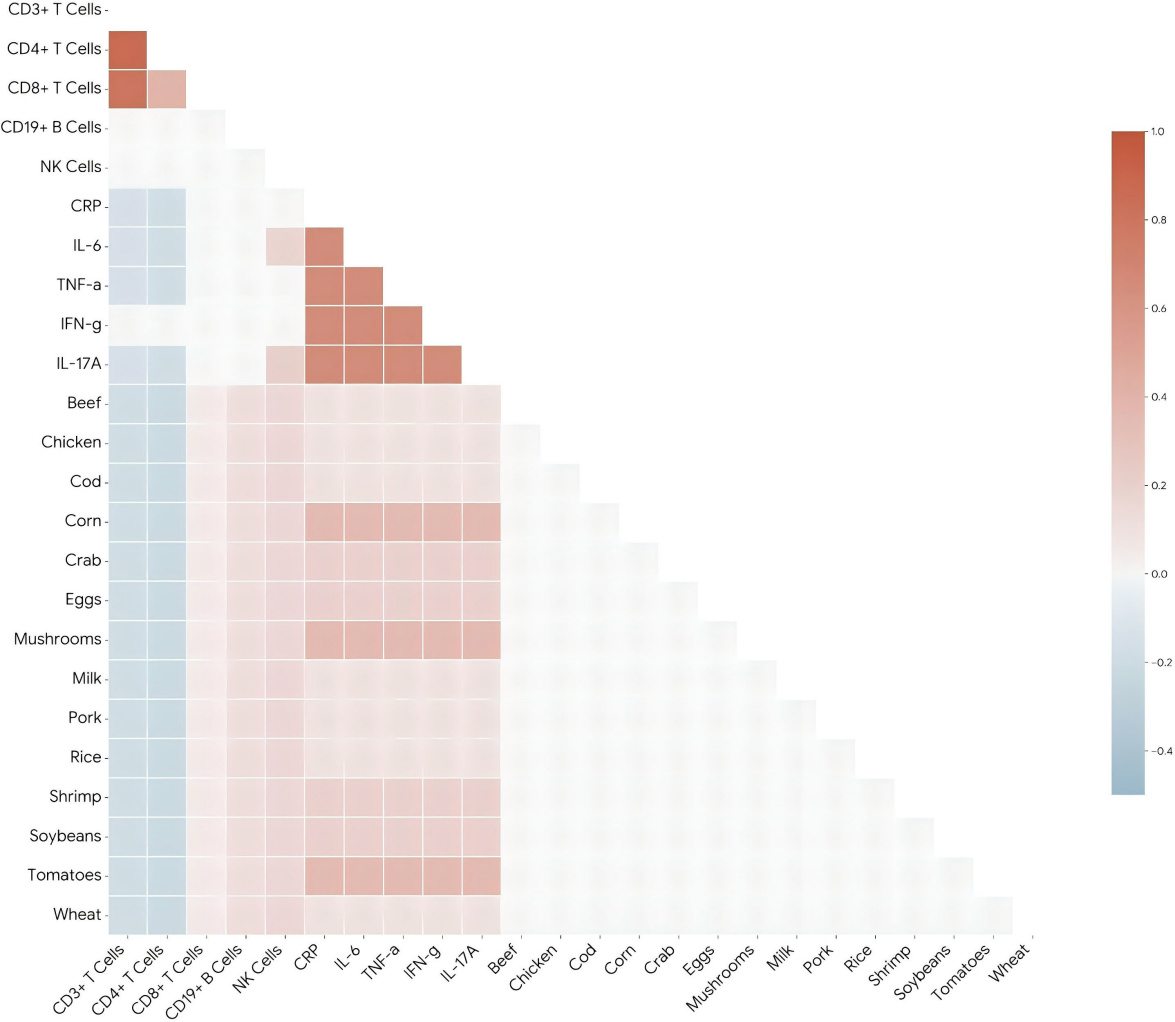
Correlation matrix of immune, inflammatory, and dietary parameters. A heatmap visualization of Spearman correlation coefficients between peripheral lymphocyte subsets (T cells, B cells, NK cells), serum inflammatory cytokines (CRP, IL-6, TNF-α, IFN-γ, IL-17A), and serum IgG antibody levels against 14 common food antigens.

There were two distinct functional clusters. First, a “pro-inflammatory dietary cluster” was identified, characterized by strong positive correlations between specific food IgG antibodies—particularly tomatoes, corn, and mushrooms—and key inflammatory cytokines, most notably IL-6 (r = 0.24, p<0.001) and IL-17A. This cluster aligns with the erosive phenotype, suggesting that these specific antigens may drive systemic inflammation. Second, a “tolerance-loss pattern” was evident, marked by a significant negative correlation between CD4+ helper T cell counts and total food-specific IgG levels (r = -0.21, p<0.001). This inverse relationship supports the hypothesis that compromised T-cell immunity facilitates the breach of oral tolerance. Unlike T cells, NK cells and B cells exhibited a positive correlation with food IgG burden (r = 0.16 and r = 0.12, respectively), indicating a divergent immune response pathway involving innate and humoral activation in the presence of food antigens (**Figure 4**).

## Discussion

**Figure 5:**
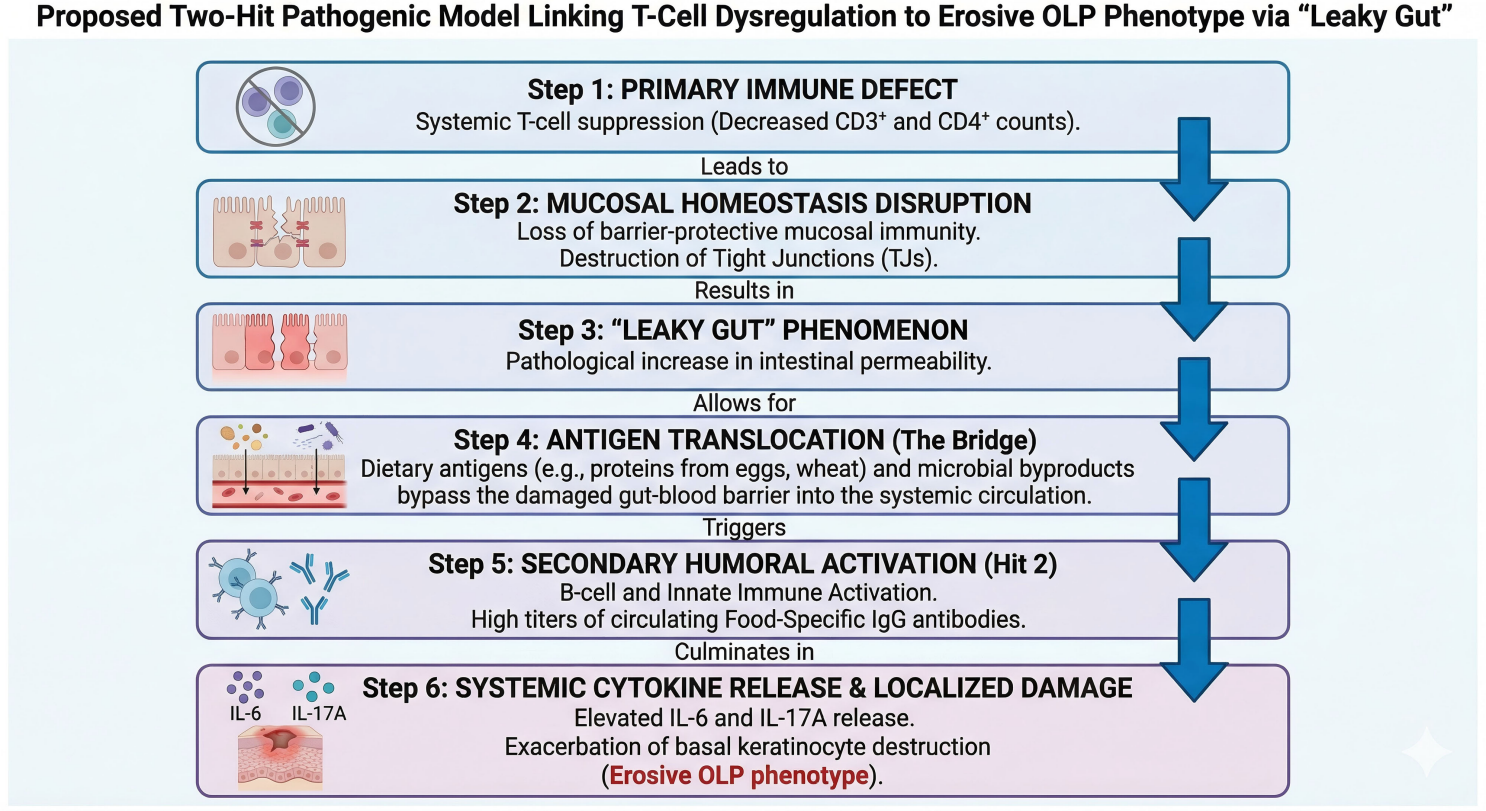
Proposed “Two-Hit” pathogenic model linking systemic T-cell dysregulation to the erosive oral lichen planus (OLP) phenotype via intestinal barrier dysfunction (“leaky gut”).

Our study challenges the traditional view of OLP as a strictly localized mucocutaneous disorder. By characterizing the immune phenotypes of 696 patients, we have identified a distinct systemic pathology characterized by cellular immune suppression and secondary food-specific humoral activation. The strong correlation between profound T-cell depletion, elevated food-specific IgG titers, and the erosive phenotype supports a novel “Two-Hit” pathogenic model (**Figure 5**). In this model, an initial defect in cellular immunity (Hit 1) compromises mucosal barrier surveillance, facilitating the ingress of dietary antigens that provoke a secondary, damaging IgG-mediated inflammatory response (Hit 2).

The hallmark of OLP pathogenesis has long been considered T-cell mediated cytotoxicity; however, our results highlight a paradoxical systemic depletion of T-cells. We observed significant reductions in peripheral CD3+ and CD4+ T-cells, particularly in patients with erosive disease (E-OLP). This finding aligns with recent work by Mao et al. (7) and Zhao et al. (8, 9), who reported that peripheral lymphocyte subsets are frequently dysregulated in OLP, potentially reflecting the recruitment of effector cells from the circulation to the oral mucosa. However, the depletion of CD4+ helper T cells suggests a broader systemic immunodeficiency rather than simple redistribution. CD4+ T cells are critical for maintaining the integrity of mucosal barriers, including the gut-blood barrier. Their deficiency is known to increase intestinal permeability (“leaky gut”). We posit that the systemic T-cell suppression observed here creates a permissive environment for antigen translocation. This concept is supported by Chen et al. (10), who demonstrated that dysregulated T-cell subsets impair mucosal homeostasis, leading to persistent inflammation. Furthermore, the age-related increase in NK cells we observed—contrasted with T-cell senescence—may represent a compensatory innate immune shift, a phenomenon also noted in aging studies (11). Recent literature also highlights that a delicate balance of CD4^+^ T cells, particularly regulatory T cells and T-helper 17 cells, is indispensable for maintaining the integrity of the intestinal epithelial barrier. A systemic depletion or functional exhaustion of these T-cell subsets compromises mucosal homeostasis, leading to a pathological reduction in tight junction proteins, such as claudins and occludins, and the upregulation of barrier-disrupting pathways (12, 13). This localized cellular immunodeficiency facilitates a mechanical barrier failure, allowing the uncontrolled translocation of undigested dietary macro-proteins and microbial antigens into the systemic circulation (14). Once systemic, these translocated antigens provoke a secondary, maladaptive humoral response (15).

The second “hit” in our model is the maladaptive humoral response to dietary antigens. We detected food-specific IgG antibodies in 57.6% of patients, a prevalence significantly higher than that reported in general populations. Unlike immediate IgE-mediated allergies, IgG-mediated responses typically operate via Type III hypersensitivity pathways, leading to the formation of circulating immune complexes that drive chronic inflammation. Cai et al. (5) previously identified similar hypersensitivity predispositions in oral mucosal disorders, suggesting that breach of oral tolerance is a key driver of chronic cheilitis and stomatitis. Crucially, our data revealed a “pro-inflammatory dietary cluster.” While eggs were the most common allergen, intolerance to tomatoes, corn, and mushrooms was specifically associated with the severe erosive phenotype. This specificity suggests that certain dietary antigens may be more immunogenic or prone to forming pathogenic complexes in the context of OLP. This aligns with findings by Veloso-Teles et al. (16), who highlighted how systemic antigenic triggers can exacerbate oral lesions through salivary and serum mediators. The significant association between female gender and IgG positivity in our cohort also mirrors the higher prevalence of autoimmunity in women described by Huang et al. (17), potentially linked to hormonal modulation of B-cell activity.

The bridge between immune complex formation and mucosal erosion appears to be a systemic “cytokine storm.” We found that patients with high food IgG titers had significantly elevated levels of IL-6 and IL-17A, which were independent predictors of the erosive phenotype. Zhang et al. (18) and Wu et al. (19) have established the critical role of the IL-6/Th17 axis in OLP, showing that these cytokines drive basal keratinocyte apoptosis and disrupt the basement membrane. Our “Two-Hit” model suggests that circulating IgG-antigen complexes stimulate myeloid cells to release these pro-inflammatory cytokines. The elevated IL-17A levels, in particular, are consistent with the “Th17 plasticity” described by Kanagaratham et al. (20), where chronic inflammation shifts the immune profile toward a destructive, autoimmune-like state. This systemic inflammatory burden likely explains why local treatments (e.g., topical corticosteroids) often fail in erosive cases; they do not address the systemic cytokine reservoir driven by ongoing dietary antigen exposure.

We identified a “High-Risk” phenotype (Subgroup A) characterized by combined immune suppression (low CD4+) and high food intolerance (high IgG). These patients exhibited the most severe clinical course, validating the synergistic impact of the two “hits.” This stratification clarifies the heterogeneity of OLP noted by Burton et al. (21), who emphasized that distinct immunophenotypes require tailored management strategies. Furthermore, Du et al. (22) and Chen et al. (23) have emphasized the importance of monitoring systemic markers to predict malignant transformation and disease progression in Chinese cohorts, a practice our risk stratification model strongly supports.

Several limitations must be acknowledged. First, the cross-sectional design prevents us from confirming the temporal sequence of T-cell depletion and IgG sensitization. While Shakoor et al. (24) suggest that systemic co-morbidities often precede oral manifestations, longitudinal studies are needed to confirm causality. Second, we did not directly measure intestinal permeability, relying instead on the established link between CD4+ deficiency and barrier loss. Future studies should incorporate markers such as zonulin, as suggested by investigations into gut-oral axis interactions (25). Third, our food-specific IgG assay detected total antibodies without distinguishing subclasses (IgG1–IgG4). Different subclasses have distinct pro-inflammatory or anti-inflammatory functions (26), which we could not evaluate in this study.

In conclusion, this study provides evidence for a systemic “Two-Hit” etiology in Erosive OLP: an underlying cellular immune deficiency facilitates a secondary food-specific IgG hypersensitivity, exacerbating a cytokine-mediated inflammatory state. These findings suggest that the current standard of care—focused solely on local immunosuppression—may be insufficient for high-risk patients. A paradigm shift toward “immunonutritional” therapy, combining systemic immunomodulation to restore T-cell competence with targeted elimination diets based on IgG testing, may offer superior remission rates for patients with refractory erosive disease.

## Data Availability

The original contributions presented in the study are included in the article/**Supplementary Material**. Further inquiries can be directed to the corresponding authors.
