# Supplementary material for: Immune dysfunction and food-specific IgG associated erosive oral lichen planus: a two-hit pathogenic model
Source: Front Immunol. 2026 May 11;17:1804758. doi: 10.3389/fimmu.2026.1804758 (PMC13199235; doi:10.3389/fimmu.2026.1804758)
Supplement: Supplementary file 1 [file Table1.doc]

### ****Supplementary Table 1: Demographic Characteristics of the Study Cohort****

| **Characteristic** | **Healthy Controls (n=200)** | **OLP Patients (n=696)** | **p-value** |
| --- | --- | --- | --- |
| ****Age (years), Mean ± SD**** | 48.5 ± 12.1 | 48.7 ± 12.3 | 0.837 |
| ****Age Group, n (%)**** |  |  | 0.921 |
| Young (18–44 yrs) | 86 (43.0) | 298 (42.8) |  |
| Middle-aged (45–59 yrs) | 76 (38.0) | 267 (38.4) |  |
| Elderly (≥60 yrs) | 38 (19.0) | 131 (18.8) |  |
| ****Gender, n (%)**** |  |  | 0.785 |
| Male | 72 (36.0) | 245 (35.2) |  |
| Female | 128 (64.0) | 451 (64.8) |  |

****Abbreviations:**** OLP, Oral Lichen Planus; SD, Standard Deviation.
****Statistical Test:**** Independent samples t-test for age; Chi-square test for age group and gender distribution.
