# Supplementary material for: Immune dysfunction and food-specific IgG associated erosive oral lichen planus: a two-hit pathogenic model
Source: Front Immunol. 2026 May 11;17:1804758. doi: 10.3389/fimmu.2026.1804758 (PMC13199235; doi:10.3389/fimmu.2026.1804758)
Supplement: Supplementary file 2 [file Table2.doc]

### **Supplementary Table 2. Age-Stratified Comparison of Lymphocyte Subset Absolute Counts Between OLP Patients and Healthy Controls**

| **Age Group / Lymphocyte Subset**  **(cells/μL)** | **OLP Patients**  **(n=696)**  **Mean ± SD** | **Healthy Controls**  **(n=200)**  **Mean ± SD** | **Mean Difference**  **(95% CI)** | **p** |
| --- | --- | --- | --- | --- |
| ****Young Adults (18–44 yrs)**** | ****(n=298)**** | ****(n=86)**** |  |  |
| Total T cells (CD3⁺) | 1050 ± 320 | 1480 ± 310 | -430 (-515 to -345) | <0.001 |
| Helper T cells (CD4⁺) | 620 ± 210 | 880 ± 240 | -260 (-315 to -205) | <0.001 |
| Cytotoxic T cells (CD8⁺) | 440 ± 190 | 590 ± 185 | -150 (-195 to -105) | <0.001 |
| B cells (CD19⁺) | 180 ± 85 | 310 ± 90 | -130 (-150 to -110) | <0.001 |
| NK cells | 135 ± 65 | 245 ± 70 | -110 (-127 to -93) | <0.001 |
| ****Middle-aged Adults (45–59 yrs)**** | ****(n=267)**** | ****(n=76)**** |  |  |
| Total T cells (CD3⁺) | 1020 ± 310 | 1400 ± 295 | -380 (-465 to -295) | <0.001 |
| Helper T cells (CD4⁺) | 610 ± 205 | 840 ± 220 | -230 (-285 to -175) | <0.001 |
| Cytotoxic T cells (CD8⁺) | 380 ± 175 | 560 ± 175 | -180 (-225 to -135) | <0.001 |
| B cells (CD19⁺) | 175 ± 80 | 290 ± 85 | -115 (-135 to -95) | <0.001 |
| NK cells | 145 ± 68 | 260 ± 75 | -115 (-133 to -97) | <0.001 |
| ****Elderly Adults (≥60 yrs)**** | ****(n=131)**** | ****(n=38)**** |  |  |
| Total T cells (CD3⁺) | 1080 ± 315 | 1350 ± 290 | -270 (-375 to -165) | <0.001 |
| Helper T cells (CD4⁺) | 640 ± 215 | 810 ± 210 | -170 (-245 to -95) | <0.001 |
| Cytotoxic T cells (CD8⁺) | 430 ± 195 | 540 ± 180 | -110 (-180 to -40) | 0.003 |
| B cells (CD19⁺) | 185 ± 85 | 270 ± 80 | -85 (-115 to -55) | <0.001 |
| NK cells | 185 ± 75 | 275 ± 80 | -90 (-120 to -60) | <0.001 |

****Abbreviations:**** OLP, Oral Lichen Planus; SD, Standard Deviation; CI, Confidence Interval; NK, Natural Killer.
****Statistical Test:**** Independent samples t-test comparing OLP patients to age-matched healthy controls within each age stratum.
