# Supplementary material for: Immune dysfunction and food-specific IgG associated erosive oral lichen planus: a two-hit pathogenic model
Source: Front Immunol. 2026 May 11;17:1804758. doi: 10.3389/fimmu.2026.1804758 (PMC13199235; doi:10.3389/fimmu.2026.1804758)
Supplement: Supplementary file 3 [file Table3.doc]

****Supplementary Table 3. Sex-Stratified Comparison of Lymphocyte Subset Absolute Counts Between OLP Patients and Healthy Controls****

| **Lymphocyte Subset**  **(cells/μL)** | **Male OLP**  **(n=245)** | **Male Controls**  **(n=72)** | **p** | **Female OLP**  **(n=451)** | **Female Controls**  **(n=128)** | **p** |
| --- | --- | --- | --- | --- | --- | --- |
| Total T cells (CD3⁺) | 1010 ± 310 | 1420 ± 305 | <0.001 | 1075 ± 325 | 1495 ± 315 | <0.001 |
| Helper T cells (CD4⁺) | 595 ± 200 | 835 ± 225 | <0.001 | 635 ± 215 | 870 ± 235 | <0.001 |
| Cytotoxic T cells (CD8⁺) | 405 ± 180 | 565 ± 175 | <0.001 | 415 ± 190 | 575 ± 180 | <0.001 |
| B cells (CD19⁺) | 175 ± 80 | 285 ± 85 | <0.001 | 185 ± 85 | 300 ± 90 | <0.001 |
| NK cells | 145 ± 70 | 255 ± 75 | <0.001 | 155 ± 72 | 265 ± 78 | <0.001 |

****Note:**** NK cells: CD3⁻CD16⁺CD56⁺. All values presented as mean ± SD.
